# Hygiene

**Published:** 1837-01

**Authors:** 


### HYGIÈNE.

*Cases of Death and Disease, produced by unwholesome Dwellings and Metallic Exhalations.* Collected by MM. D'Arcet and Braconnot.

Case i. Gillet, a cabinet maker, æt. 35, of a good constitution, lived, with his wife and three children, in a house at Nancy, which he had recently purchased. The whole family had the same symptoms as himself: pains in the head, nausea, lassitude, difficult digestion, almost constantly colic, diarrhœa, swelling and numbness of the limbs, extreme exhaustion, and depression of spirits. His workmen were not ill, nor was a woman who took her meals with him, and remained in his house during the day, but who did not sleep there. His neighbours also were not affected. M. Braconnot was requested to investigate the case. It was suspected that the water of his well might have become impure by infiltration, as his next-door neighbour, M. Noel, was a manufacturer of coloured paper, and made use of large quantities of arsenic and oxide of copper in the preparation of the green of Schweinfurt; but an accurate analysis proved that this was not the case, and that the water was wholesome. His apartments on the first floor were clean, commodious, and sufficiently open in front; but in his shop there was a large damp spot on the floor, which he said corresponded to a dark outhouse beneath, belonging to M. Noel's manufactory. On going down into it, it was found to be as dark as a cellar, only lighted with a small opening, three or four feet square in the roof. It had not been used for fifty years, but for a long time all kinds of rubbish had been thrown into it from the manufactory, through a little obscure casement. In this outhouse was a well, only a short distance from the one belonging to Gillet, and beneath his sleeping room: on letting down a candle, it burned at the surface of the water, but bubbles of gas were seen spontaneously disengaged, and they became very numerous on agitating the water by throwing in stones: it was hydrogen gas, like that in marshes, and arising from the decomposition of the organic matters thrown into the well. The water did not appear to be worse than that in stagnant marshes. This undoubtedly (says M. Braconnot,) was the cause of the disease. M. Noel, who had lived in his manufactory thirty years, informed him that, twenty-five years since, Grandidier, a robust and athletic man, died of the same disease as Gillet, in the same house; also that the wife and three children of Royer, who lived there fifteen years ago, died with similar symptoms; Madame Mathieu also, and her young daughter, two years ago, and Laurent and his two nieces, died in the same house, from the same malady. However, twelve years before, a family lived there two years without illness. M. D'Arcet thus accounts for the entrance of the noxious exhalations:—In winter, (the month of November was mentioned,) a fire is made in M. G.'s rooms; the exterior air enters the outhouse by the opening in the roof, is infected, and is drawn into the apartment by the draught of the chimneys.

Case ii. M. D'Arcet had to examine a lodging in which three young and vigorous men had died in succession in a few years. The lodging consisted of a bedchamber with a chimney, and an anteroom without ventilation. The descending pipe of a water-closet passed down the angle of the alcove, by the side of the head of the bed, and the wall in this part was slightly infiltrated: notwithstanding this, there was no sensible smell in the room, although it was small and low. M. D'Arcet could only attribute the mortality in the lodging to the slow action of the emanations from the pipe, which were, particularly during the night, drawn around the head of the bed and into the room by the draught of the chimney.

CASE III. M. D'Arcet observed the health of one of his acquaintances to languish and decline, although young and of good constitution; and he requested him often to examine his lodging, and even to quit it: at last his friend besought him to investigate the cause of the uneasiness he felt whenever he remained at home. M. D'Arcet found that the room was often filled with the gaseous products of the combustion of carbon. The chimney of his parlour, in which he seldom had a fire, was common to a kitchen on the story above; and the carbonic acid gas, which descended by the chimney of the parlour, was drawn into the bedroom by the draught always kept up in its chimney at night, by a fire in winter, and by the heat of the room (which was small,) in summer. The cause of the evil being known, it was easily remedied by making a good chimney in the bedroom, placing a trap-door in the chimney of the parlour, and also sand-bags at the door separating the two rooms.

CASE IV. A whole family was salivated, they knew not how. They recollected at last that a barometer had been broken, and the mercury put in a plate, which was placed in a cupboard. This was removed, and the disease disappeared. The least ventilation would have prevented so slight a cause from producing such severe effects.

CASE V. A prefect of police requested M. D'Arcet, at six o'clock in the morning, to examine a room in which two females had been asphyxiated during the night. He easily recognized the presence of carbonic acid, which he found had entered the bedchamber by the stove of the dining-room, where no fire had been made for a long time; that it had traversed the dining-room, and had penetrated the bed-room owing to the draught of the bed-room chimney. The owner of the house, on being questioned, said that the chimney into which the pipe of the stove passed belonged to the room of a dentist who occupied the first floor. M. D'Arcet knocked at the dentist's door, and he opened it himself: he had his pincers in his hand, and had been spending the night in making artificial teeth, for which he used a furnace heated with charcoal, and had thus asphyxiated the two ladies lodging above him.

CASE VI. A whole family became ill from the vapour of mercury, issuing from the workshop of a gilder. The chimney of the stove in their lodgings communicated with the chimney of the gilder, and a draught from a chimney in the rooms of the former drew the mercurial vapour through the chimney of the stove, and diffused it.

M. D'Arcet says he could recite many other instances from his own knowledge: these show that care should be taken in choosing the place from whence the air is drawn for the necessary draught of the chimneys of an apartment, which requires plenty of pure air, and not air from an infected place, such as the tube of a water-closet, an unwholesome court, neighbouring chimneys, &c. The least ventilation which is constant, whether from below upwards or from above downwards, is sufficient to purify a lodging, but a constant stream of pure air is necessary, cold in summer, and hot in winter.

CASE VII. Whilst M. Vauquelin lived at l'Ecole des Mines, his house was kept by two sisters of Fourcroy. These ladies, who kept a dog, cat, and canary birds, went to spend two days in the country with M. Vauquelin; gave the animals plenty to eat and drink, and shut them up in the antechamber. M. Vauquelin found, on his return, the antechamber full of smoke, and the animals dead: the smoke had penetrated into the apartment by the tube of the stove, and came from a chimney on the upper story. The smoke had either fallen when it cooled, or it had been brought into the room by the draught of a chimney of M. Vauquelin's, the tube of which might have been heated either on the roof by the sun, or by its proximity with a neighbouring chimney in which there might have been a fire.

CASE VIII. M. Berthier was requested to attend a man named Salomon, his wife and child, who were all violently salivated; as also a young workman, who worked during the day with Salomon. It appeared, on enquiry, that M. Husson, a gilder, lived in the story below, and his chimney was the same as Salomon's. With this chimney M. Husson's furnaces communicated: he was in the habit of placing on the furnaces the articles he manufactured, in some of which mercury was used; the action of the fire volatilized the mercury, which entered the chimney, and was drawn by the draught into the pipe of Salomon's stove, where it was deposited, to be again volatilized when the stove was heated; the vapours then were diffused through the room, and produced the serious effects mentioned. To test the presence of mercury, a piece of gold was rubbed against the sides of the stove, which was at once silvered. To remedy this the stove was removed, and the hole in the chimney stopped: prudence probably demanded that the chimney itself should have been condemned.

[The perusal of this paper will be of some advantage if it directs attention to an important set of causes of disease, which are too often neglected by the medical practitioner, who directs his sole attention to the cure of the actual malady. The cause of profuse salivation of a whole family is not likely to be overlooked; but less prominent, though not less important, cases of impaired health from various noxious exhalations very frequently are, as we know from experience. It should be recollected that these cases happened in France, where numerous families live in one house; stoves are very frequent, and charcoal much employed in heating them.]

*Annales d'Hygiène, Juillet,* 1836.

